# A comprehensive analysis of two types of xenogeneic bone particles for use in maxillofacial bone regeneration therapies

**DOI:** 10.1371/journal.pone.0323754

**Published:** 2025-05-19

**Authors:** Olimpia Ortiz-Arrabal, Mario Anibal Rodriguez, Jesús Chato-Astrain, Miguel Ángel Martín-Piedra, Ingrid Garzón, Víctor Carriel, Ricardo Fernández-Valadés, Antonio España-López, Miguel Alaminos, Ismael Angel Rodriguez

**Affiliations:** 1 Tissue Engineering Group, Department of Histology, School of Medicine, University of Granada, Granada, Spain; 2 Instituto de Investigación Biosanitaria ibs.GRANADA, Granada, Spain; 3 Department of Histology, Embryology and Tissue Engineering, School of Dentistry, National University of Cordoba, Cordoba, Argentina; 4 Craniofacial Malformations and Cleft Lip and Palate Management Unit, University Hospital Virgen de las Nieves, Granada, Spain; 5 Division of Pediatric Surgery, University Hospital Virgen de las Nieves, Granada, Spain; 6 Department of Stomatology, School of Dentistry, University of Granada, Granada, Spain; Universidade de Trás-os-Montes e Alto Douro: Universidade de Tras-os-Montes e Alto Douro, PORTUGAL

## Abstract

Regeneration of maxillofacial bone structures is challenging. One strategy for bone damage repair involves using bone filler particles. This study analyzed the regenerative potential of deproteinized bone particles (DP) and collagen-based bone particles (CP) to determine the effectiveness of each biomaterial in bone repair. Structural analysis using scanning electron microscopy and 3D scanning showed that DP and CP were structurally similar, comprising a heterogeneous mixture of bone particles of varying sizes and shapes. Ex vivo analyses, including morphological evaluation, LIVE & DEAD assays, and DNA quantification, demonstrated high biocompatibility of CP and DP with human cells in both direct and indirect contact at 24, 48, and 72 hours. Both particles were grafted onto Wistar rats with a critical mandibular defect for two months. Computed tomography revealed significant defect reduction in the CP group, but not in the DP group, compared to negative controls without any bone particles. Histological analysis showed biocompatibility of both particles in vivo and identified regenerative tissue with collagen fibers and mineralized spots in CP and DP, with more mineralized spots in DP. Histochemistry and immunohistochemistry confirmed collagen, proteoglycans, and osteocalcin presence in the regeneration area of CP and DP. These results confirm the biocompatibility and potential of both particle types for maxillofacial bone regeneration, particularly CP. Future studies should assess their clinical usefulness for patients with cleft palate, mandibular damage, and other maxillofacial applications involving tissue engineering techniques.

## Introduction

Bone regeneration therapies applied to palate and mandible bone defects is one of the research areas in need of novel therapies able to improve current results [1–4]. In this regard, the recent development of tissue engineering allows the development of novel therapies based on three fundamental components: cells, biomaterials, and growth and inductive factors [5,6]. Application of tissue engineering allowed the generation of several tissue substitutes with potential clinical usefulness, including some models of the human bone [7]. In addition, guided bone regeneration (GBR) is a surgical technique that relies on grafts and barrier membranes for bone regeneration [8,9]. Typically, bone grafts are used as osteoconductive or osteoinductive biomaterials, whereas barrier membranes play a role as osteopromotive biomaterials able to improve the regenerative results of this technique to re-establish bone function and aesthetic [10,11].

One of the most common osteoconductive biomaterials used in guided bone regeneration are the bone filler particles. Bone filler particles are commonly obtained from different origins, such as the own patient (autologous particles), human donors (allogeneic), other animal species (xenogeneic) and several synthetic sources (alloplastic) [12,13]. The choice of a specific biomaterial depends on various factors, and there is no universally applicable biomaterial for all clinical scenarios. Furthermore, it is essential to recognize that all available bone filler particles have their drawbacks. For instance, autologous bone particles, derived from the patient’s own bone, offer the advantage of being both osteoinductive and osteoconductive. However, their main disadvantage lies in the need for a donor site, which results in postoperative morbidity. Additionally, there is a limitation in the quantity of available filler material, and its biodegradability lacks predictability.

To overcome these disadvantages, xenogeneic particles are now available as a valid alternative [12–14] that can be combined with exosomes [15] or hydrogels [16]. The xenogeneic particles currently employed are mostly obtained from porcine and bovine sources. These materials are chosen due to their chemical composition and histological structure, which closely resemble those of human bone [12]. Different studies have shown that these particles have good osteoconductive properties, since they behave like good matrices or scaffolds where cells can settle, proliferate, differentiate and synthesize key products for the formation of bone tissue [13,17]. In most cases, bone particles can be manufactured using different biofabrication methods, which results in the generation of particles with different compositions [18]. In general, most xenogeneic bone particles consist of a decellularized hydroxyapatite matrix that can contain collagen (collagen-based particles or CP) or a deproteinized hydroxyapatite matrix from which collagen has been removed (deproteinized particles or DP) [13]. These variations in bone particle composition and structure can lead to variable *in vivo* behavior, different biodegradability and different amount of newly-formed bone at the regenerating area [17].

Although new types of CP and DP have been recently described and are currently used in patients [18], the definite indications of each type of particle have not been established, and determining the functional and structural properties of each type of particle applied to bone regeneration is in need. In fact, a proper histological analysis able to elucidate the behavior of these biomaterials concerning biocompatibility, biodegradability, and their role in the bone regeneration process is essential for selecting an appropiate biomaterial for clinical use based on the specific therapeutic context to be addressed. In this work, we carried out a comparative *ex vivo* and *in vivo* analysis of two xenogeneic bone filler particles (CP and DP), obtained by using different processsing treatments, to shed light into their biocompatibility, biodegradability and functional properties in the process of bone regeneration.

## Materials and methods

**Fig 1:**
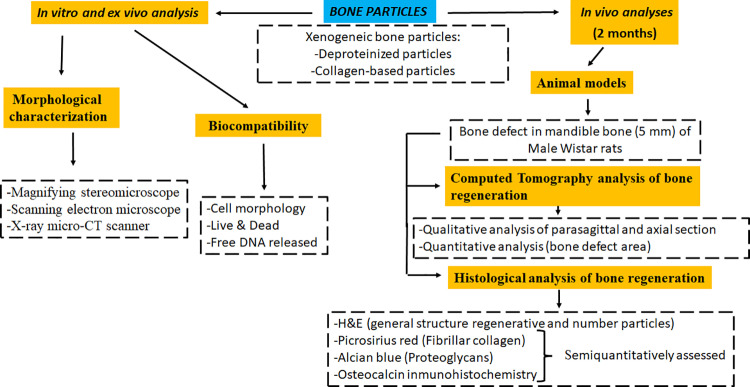
Methods used to evaluate the two types of xenogenic bone particles compared in the present work. The different methods and procedures performed in this work are summarized in a flow chart.

Fig 1 summarizes the methods used in this study which are detailed below.

### Bone particles and *in vitro* morphological characterization

In this study, we compared two types of xenogeneic bone particle fillers commercialized for clinical use. First, we used deproteinized particles (DP) consisting of a mineral matrix of hydroxyapatite of bovine origin that was subjected to deproteinization (BOS-HA EVOLUTION, TISSUM^®^ Biomateriales, Córdoba, Argentina). Then, we used collagen-based particles (CP) composed of a bone matrix of collagen and hydroxyapatite of porcine origin (SUS-OSS, TISSUM^®^ Biomaterials, Córdoba, Argentina). In both cases, the bone particles were obtained from the cortico-cancellous bone of the femoral heads and condyles of animal bones and had a granular appearance with a particle size ranging from 200 to 1000 micrometers.

To perform a morphological characterization of each type of bone particles, CP and DP were first observed using a magnifying stereomicroscope Nikon SMZ 745T (Nikon Instruments Inc., Tokyo, Japan). Then, samples were dissecated, covered with gold-paladium atoms, and analyzed using a microscope FEI Quanta 200 scanning electron microscope (FEMINI, Carl Zeiss SMT, Germany) using the high vacuum mode. In addition, both types of particles were analyzed using a high-resolution Xradia 510 VERSA X-ray micro-CT scanner (ZEISS, Oberkochen, Germany). For this, particles were inserted in an analysis tube transparent to the X rays, and scanned at high resolution with a voxel size of 70nm. Exposition parameters were 80 kV and 7W, with a pixel size of 0.63 per image. Tomographic sections were obtained, and 3D reconstructions were generated from each type of particle.

To determine the porosity grade of each type of particle, sections were analyzed using ImageJ (National Institutes of Health, USA) automatic quantification, as previously reported [19]. In brief, images were converted to binary, and 15 random areas of 200µm × 200µm were selected and automatically analyzed to determine the area fraction corresponding to empty spaces (osteocytes lacunae) in each type of particle, corresponding to the percenteage of porosity.

### Analysis of *ex vivo* biocompatibility of CP and DP bone particles

Human gingival fibroblasts were obtained from oral mucosa biopsies and cultured for 24 hours in 24-well plates (2 × 10^4^ cells/well) using Dulbecco’s Modified Eagle’s Medium (DMEM) with 10% Fetal Bovine Serum (FBS) and a commercial antibiotics/antimycotics cocktail solution (all cell culture reagents from Merck, Burlington, MA, USA) at 37°C with 5% of CO_2_. These human oral mucosa biopsies were recruited from March 1^st^, 2023 to June 30^th^, 2023, and all participants provided written informed consent for their participation in the study. Two experimental methods were used to evaluate the effect of both types of bone particles on the human cells, as previously reported [20]. In the first method (direct contact or DC), 10 mg of hydrated bone particles were added per well containing the cultured cells, to allow the particles to directly contact the cells. In the second method (indirect contact or IC), the same concentration of bone particles was added on the membrane of a Transwell^®^ culture insert (Costar-Corning, Corning, NY, USA) used to separate the culture cells and the particles, allowing the culture medium to contact both things through the pores of the polyester membrane of the inserts. Previous studies demonstrated that any biologically active factors released from biomaterial could freely flow through the membrane and make contact with the cells [21]. Subsequently, 1,500 µl of culture medium were added in both cases (DC and IC) to completely cover the human cells and the particles. As positive controls of live cells (CTR+), cells were cultured in their regular culture medium, without any added particles. As negative controls (CTR-), cells were incubated with 1% triton X-100 (PanReac AppliChem, Barcelona, Spain), able to induce cell death of all cultured cells.

Biocompatibility was determined in the DC and IC groups and controls after 24, 48 and 72 hours using a combination of cell viability and function tests, as previously described [22,23]:

1) Cell morphology of cells cultured with CP and DP bone particles was analyzed using phase contrast microscopy.
2) Live & Dead (LD) dual fluorescence assays (Invitrogen, Eugene, Oregon, USA) were used to distinguish between living and dead cells. As a dual functional and structural method, LD contains calcein-AM, which is metabolically modified by functional cells to produce a green pigment, and ethidium homodimer-1, which stains the nuclei of dead cells with membrane structural damage, in red. Images were taken from each experimental condition using a Nikon Eclipse 90i fluorescence microscope (Nikon), and the percentage of green (live) and red (dead) cells was calculated. Results were normalized to the CTR+ (considered as 100% cel viability), and CTR- (considered as 0% cell viability).
3) Free DNA released to the medium as a consequence of irreversible cell damage was quantified from the culture medium using a NanoDrop 2000 UV–vis spectrophotometer (Thermo Fisher Scientific, Waltham, MA, USA). Results were normalized to the CTR+ (considered as 100% cel viability), and CTR- (considered as 0% cell viability).

### *In vivo* analyses

#### Animal models.

To evaluate the effects of CP and DP particles on bone regeneration, both types of particles were implanted in laboratory animals in which a bone defect had been generated at the mandible bone. Male Wistar rats weighing 250-300g maintained in the Experimental Unit of the University Hospital Virgen de las Nieves in Granada (Spain) were used. Animals were housed in a controlled temperature room (21 ± 1°C) using a 12 h light/dark cycle with ad libitum access to tap water and rat chow. These studies were performed according to the European Union and Spanish Government guidelines for the ethical care of animals (EU Directive No. 63/2010, RD 53/2013). Animals were deeply anesthetized by intraperitoneal injection of acepromazine (Calmo-Neosan^®^, 0.001 mg/g, Boehringer Ingelheim, Ingelheim am Rhein, Germany) and ketamine (Imalgene 1000^®^, 0.15 mg/g, Boehringer Ingelheim). Then, the left side of the mandible of each animal was surgically exposed by separating the muscles at the angle of the mandible to expose the mandibular bone. After that, a bone defect of 5 mm in diameter was generated at the mandibular angle using a trephine, based on previous works demonstrating that this defect corresponds to a critical-size defect in this experimental model [24,25]. The following experimental groups were established (n = 5 per each group):

1. Native: Animals that had not been subjected to any surgical procedure used as positive controls.
2. Negative control (NEG): In this group, the critical bone defect was not filled with any material. This group was considered as a negative control of bone regeneration.
3. CP: In this group, the bone defect was filled with CP particles.
4. DP: In this group, the bone defect was filled with DP particles.

In the NEG, CP, and DP groups, the muscles were reintegrated to their original site, and the skin and soft tissues surgical defect was repaired using absorbable suture material. In all animals, analgesia was provided with metamizole (Boehringer Ingelheim) diluted in drinking water for 5 days after the surgical procedure.

Animals were euthanized after 2 months of follow-up using a euthanasia solution (Eutanax, Fatro Ibérica S.L., Barcelona, Spain) under general anesthesia. In order to alleviate animal suffering, humane endpoints were established for all animals involved in the study. In case any of the animals displayed any early marker of suffering, poor quality of life or distress, such as abnormal behavior, body temperature or weight changes, tumor size or appearance, pathological changes, ruffled fur, reduced mobility or abnormal body postures, the animals would be immediately euthanized under general anesthesia. These signs were monitored by expert personnel with experience in animal care and welfare every day during the 2 months in which the experiment was carried out. At the end of the follow-up period, none of the animals fulfilled these humane endpoint criteria, and none of the animals died before this follow-up time. This project was approved by the CEEA ethical committee for animal experimentation (approval number: 03-7-15-311).

#### Computed Tomography analysis (CT).

Immediately following euthanasia, animals were analyzed using a PointNix Point 3D Combi 500C Dental Imaging System^®^ (Abex Medical System, Selangor Darul Ehsan, Malaysia). The heads of the animals were placed and secured on the analysis surface of the analysis device, and high-resolution images were scanned from each animal. 3D reconstruction images were then generated, and the defect site at the left side of the mandible was analyzed. Samples were scanned using 266 mAs, at 14 mA and 70 kV. Bone regeneration in each experimental group was evaluated through qualitative analysis of parasagittal and axial radiographical sections.

Additionally, a quantitative bone regeneration analysis was performed by determining the area of the bone defect in each experimental group, after the established 2-months follow-up time, using parasagittal plane sections. Results obtained in the CP and DP groups were normalized to the NEG group, whose bone defect area was considered as 100%.

#### Histological analysis of bone regeneration.

To perform a structural and histological analysis of bone regeneration, the mandibular region of the specimens was fixed in 4% formalin, decalcified in formic acid-sodium citrate (Ana Morse) reagent for 14 days, embedded in paraffin and cut into 5 μm sections in a frontal plane through the middle zone of the critical bone defect. To determine the histological structure and analyze morphology of the defect site, tissue sections were deparaffinized, rehydrated and stained with hematoxylin and eosin (H&E) following routine histological methods. In each animal, the general structure of the bone and the presence of any regenerative tissue were analyzed, and the number of particles present at the bone defect was quantified using areas of 160 mm^2^. The presence of fibrillar collagen was assessed using picrosirius red (PSR) histochemical staining [4,26], while proteoglycans were detected using alcian blue (AB) histochemistry [4]. Then, expression of the bone extracellular matrix protein osteocalcin (OCC) was revealed by immunohistochemistry following routine protocols [4]. For the histochemical and immunohistochemical analyses, the signal intensity was semiquantitatively assessed as strongly positive (+++), mildly positive (++), slightly positive (+) or negative (−), as previously described [19].

### Statistical analysis

First, each variable was analyzed using the Shapiro-Wilk test of normality. As most variables turned out to be non-normally distributed, non-parametric statistic tests were then used. Results of the analysis of porosity of CP and DP bone particles, and the number of mineralized particles found in the *in vivo* experiments were compared between both groups using the test of Mann-Whitney. For the *ex vivo* analysis of cell viability (LD assay and DNA quantification), differences with the positive control group (CTR+) were evaluated using the Exact Test of Fisher, as results were expressed in percentages. To compare the area of the defect found in the *in vivo* experiments determined by CT scanning, we first calculated the average size of the bone defect in each study group, normalized to the NEG animals (considered as 100% of defect size). Then, percentages in each group were compared to the NEG group using the Exact Test of Fisher. All tests were carried out two-tailed. To prevent errors associated with multiple testing, statistical p values below 0.001 were considered statistically significant. Statistical comparisons were carried out by using the Real Statistics Resource Pack software (Release 7.2) (Dr. Charles Zaiontz, Purdue University, West Lafayette, IN, USA), available at www.real-statistics.com.

### Ethics statement

This study was approved by the regional ethics committee CCEIBA (Comité Coordinador de Ética de la Investigación Biomédica de Andalucía), ref. 2044-N-22 (date of approval 13^th^, February 2023) and ref. 1961-N-19 (date of approval 28^th^, November 2019). All tissue donors provided written informed consent to participate in the study. Animal experimentation was approved by the Ethics Committee for Animal Experimentation in Andalusia (CEEA), protocol code 08/07/2019/123 (date of approval 10^th^, September 2019) and 08/07/2019/122 (date of approval 26^th^, July 2019).

## Results

### Bone particles characterization

**Fig 2:**
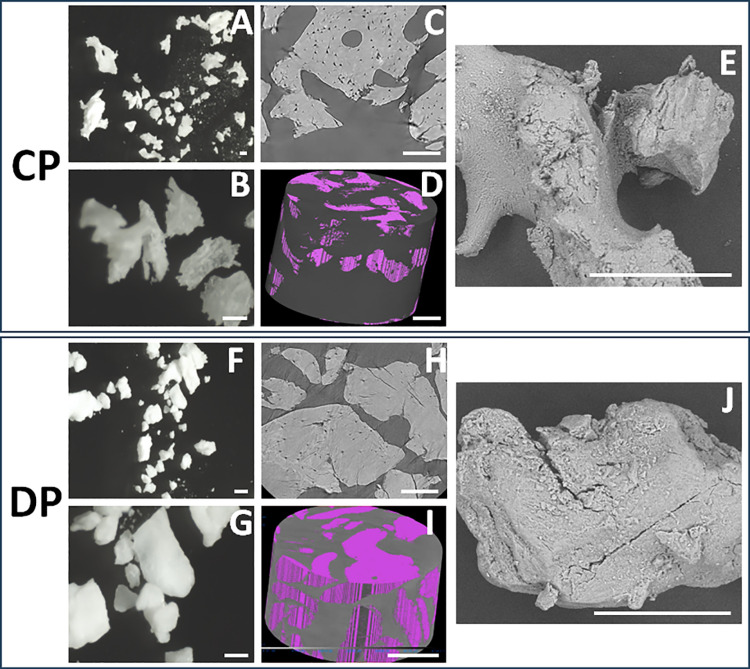
Morphological characterization of both types of bone particles. CP (collagen-based particles) and DP (deproteinized particles) were analyzed using a stereomicroscope (A, B, F, G), a scanning electron microscope (E, J), and a micro-CT scanner (C, D, H, I). Video images corresponding to panels D and I are available as S1 and S2 Files, respectively. Scale bar: 200µm.

Morphological evaluation of CP and DP bone particles (Fig 2 and S1 and S2 Files) using a stereomicroscope showed that both types of materials consisted of a heterogeneous mixture of bone particles of different sizes and shapes. When the particles were analyzed with scanning electron microscopy, we found that both types of particles were irregular and showed holes and depressions of different depths and sizes. Analysis of CP and DP using micro-CT scanning revealed the presence of small cavities inside both materials, corresponding to lacunae of osteocytes. Analysis of porosity showed that 3.76 ± 3.18% of CP particles and 4.83 ± 2.67% of DP corresponded to porous areas, with non-significant differences between both types of particles (p > 0.05).

### *Ex vivo* analysis of biocompatibility

**Fig 3:**
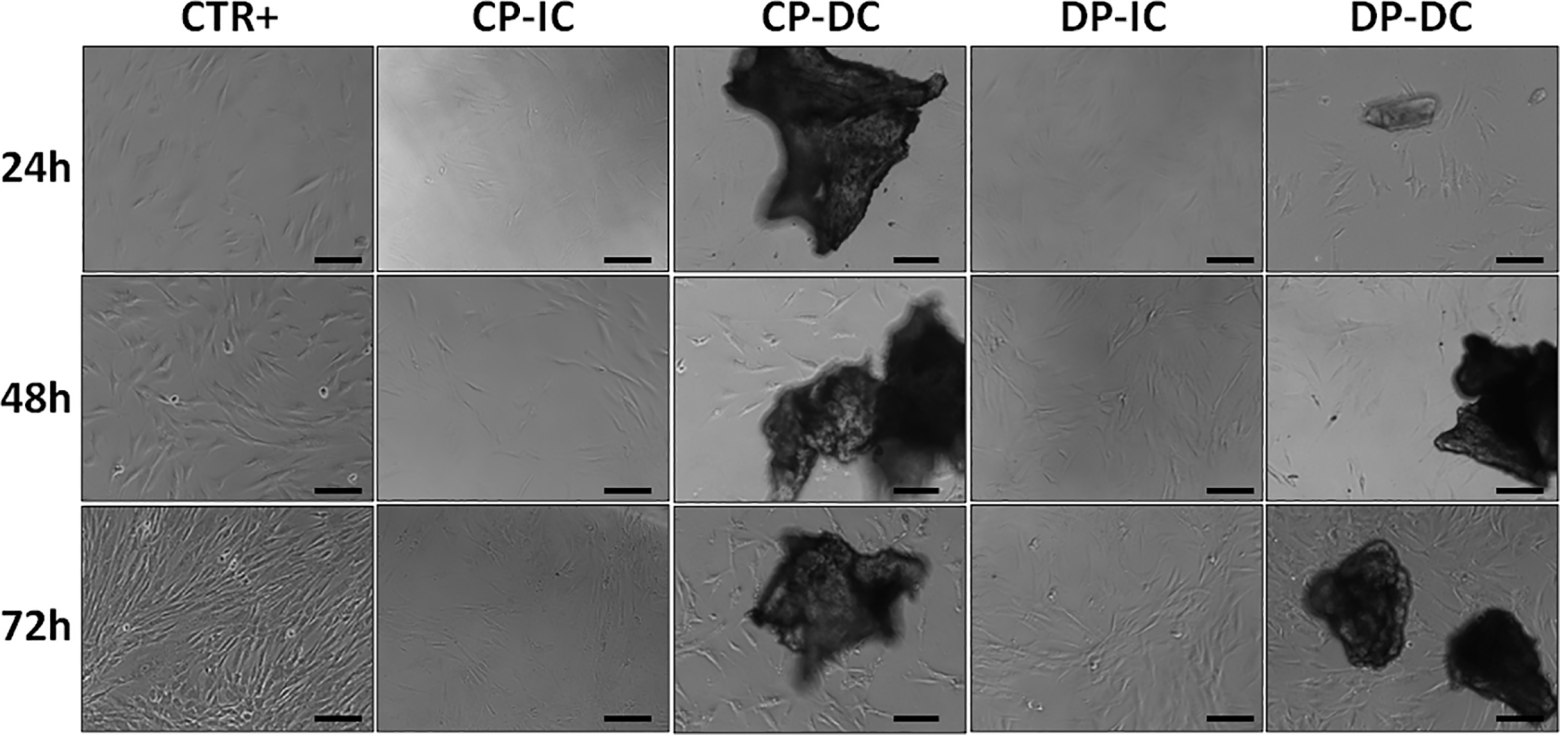
Morphological analysis of human cells cultured with CP and DP using phase-contrast microscopy. Illustrative images are shown of human cells cultured for 24, 48 and 72h in direct contact (DC) or indirect contact (IC) with collagen-based particles (CP) or deproteinized particles (DP). Scale bars: 100 µm.

On the one hand, the morphological analysis of cells cultured in the presence of CP and DP bone particles showed that cells retained the typical elongated, spindle-like shape of viable human fibroblasts in both the DC and IC groups, similar to CTR+ cells cultured in the absence of bone particles (Fig 3). No morphological differences were observed among the different culture times, although the number of cells tended to increase with time.

**Fig 4:**
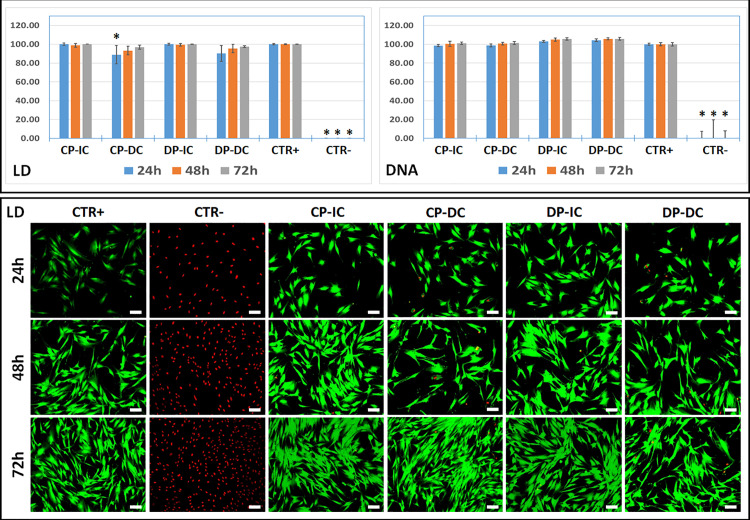
Analysis of cell viability using Live & Dead assay (LD) and free DNA quantification (DNA). The top panel corresponds to histograms representing the quantitative values obtained for each analysis method in each study group and controls, after 24, 48 and 72h of follow-up, shown as percentages of cell viability normalized to controls. The lower panel shows illustrative images of cells analyzed with LD. Live cells appear in green, whereas dead cells are stained in red. DC: direct contact; IC: indirect contact; CP: collagen-based particles; DP: deproteinized particles; CTR + : positive control of cells cultured without bone particles (live cells); CTR-: negative control of cells treated with 1% triton X-100 (dead cells). Statistical differences with the CTR+ group are labeled with asterisks in the histograms (*). Scale bars: 100 µm.

On the other hand, cells were evaluated using the LD assay (Fig 4 and S1 Table), which assesses metabolic function and membrane integrity. For both the DC and IC assays, our results showed high cell viability in cells cultured with CP and DP, with non-significant differences with CTR + , except for the CP group at 24h with DC. In this case, a significant decrease in viability was observed, although the viability remained above 88%, and normalized after 48 and 72h. Viability was also analyzed at the structural level by measuring the amount of DNA released from cells cultured with CP and DP. Results showed that cell viability remained above 97% at all times in the CP and DP groups (DC and IC), with non-significant differences compared to the CTR+ group. In contrast, the CTR- group showed a significant decrease in cell viability compared to all the other groups, at the three follow-up times (Fig 4 and S1 Table).

### *In vivo* functional analysis of mandibular bone regeneration

**Fig 5:**
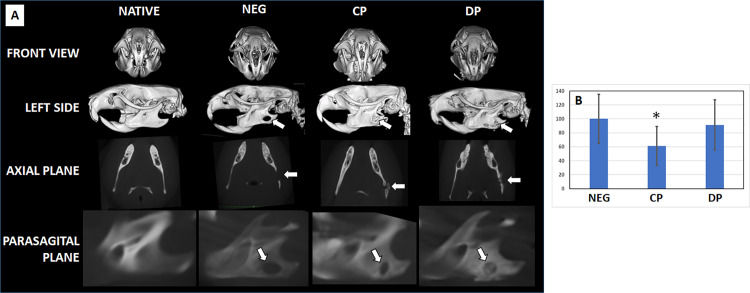
CT scanning analysis of the craniofacial bones of animals included in the in vivo study. A: CT images obtained in different planes (front view, left side, axial plane, and parasagittal plane). B: Size of the mandibular bone defect normalized to the negative control group (NEG), considered as 100% of the defect size. Results are shown as means and standard deviations, and significant differences with the negative control group are highlighted with asterisks (*). Native: positive control of non-operated animals; NEG: negative control group of animals subjected to bone defect devoid of any bone particles; CP: animals subjected to bone defect filled with collagen-based particles; DP: animals subjected to bone defect filled with deproteinized particles. The bone defect is labeled with white arrows in panel A.

When CP and DP particles were evaluated in a rat model of mandibular bone defect, we found some differences among groups. As shown in Fig 5, we first found that the CT image of the mandible of animals corresponding to the Native group was compatible with a normal bone, and no defect was found. In contrast, NEG animals showed a large mandibular defect after 2 months of follow-up. Regarding the experimental group in which CP bone particles were implanted, we found a significant reduction of the bone defect area as compared with NEG (p < 0.0001), with a defect area in CP corresponding to 60.92 ± 28.19% of the NEG defect. Finally, analysis of the bone defect in the DP group revealed a slight reduction in the area of the bone defect, which corresponded to 92.10 ± 36.13% of the NEG group, with differences between DP and NEG being non-significant (p = 0.0067). Differences between the CP and the DP groups were statistically significant (p < 0.0001). In addition, the qualitative analysis of the CT scan results revealed that the defect site in the DP group was occupied by radiopaque structures consistent with bone particles, as seen in the axial and parasagittal planes. However, these structures were not found at the defect site in the CP group. Interestingly, the antero-posterior edges of the bone defect were found to be closer in the CP group as compared to the NEG and DP groups. Furthermore, the referred bone defect edges were denser in the CP and DP groups than in the NEG group.

### Histological analysis of bone regeneration

**Fig 6:**
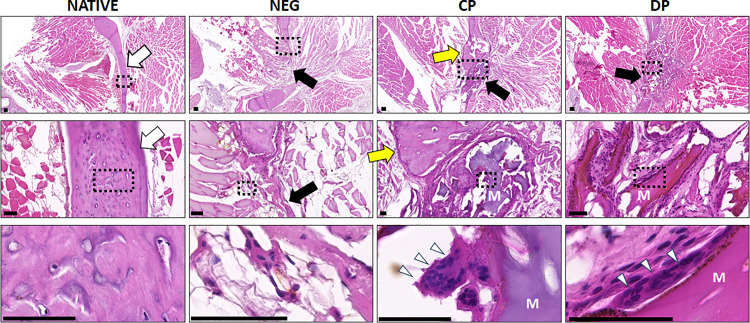
Histological analysis of animals grafted with the different types of bone particles and controls using hematoxylin and eosin staining (H&E). Images obtained from each study group are shown at different magnifications, with images at higher magnifications corresponding to the square inserts in the images above. Native: positive control of non-operated animals; NEG: negative control group of animals subjected to bone defect devoid of any bone particles; CP: animals subjected to bone defect filled with collagen-based particles; DP: animals subjected to bone defect filled with deproteinized particles. White arrows point to illustrative areas of native mandibular bone; Yellow arrows label the dilated area of bone at the edges of the defect; Black arrows point to the critical bone defect; Giant cells are labeled with white arrowheads; M: mineralized structures. Scale bar: 200 µm for the top panel and 50 µm for the medium and lower panels.

The histological analysis of the mandibular region corresponding to the different experimental groups allowed us to evaluate mandible bone regeneration after 2 months of follow-up. As shown in Fig 6, our analysis using H&E staining showed that the mandible bone of Native non-operated animals consisted of a compact lamellar tissue containing abundant osteocytes, compatible with a normal bone. In contrast, the NEG group revealed the presence of a bone defect devoid of bone tissue that was filled with a soft, connective tissue containing fibrous bundles and few cells. When the bone defect was filled with biomaterials in the CP and DP groups, we evidenced that the defect area contained a denser connective tissue whose volume was higher than that found in the NEG group. This tissue consisted of abundant fibers and cells, along with dense, mineralized structures that could correspond to the grafted particles. When the number of mineralized structures was quantified, we found that the DP group contained higher number of these structures than the CP group (3.5 ± 1.3 vs. 1.5 ± 1.0), with differences being statistically significant (p < 0.001), and these structures were more heterogeneous and more basophilic in the DP group. In addition, we found that the edges of the bone defect were dilated, forming a widened structure, in both the CP and DP group, especially in CP. In both cases, abundant multinucleated giant cells were observed surrounding the mineralized structures. No signs of complications or side effects, such as tumorigenesis, rejection or necrosis were observed in any of the animals.

**Fig 7:**
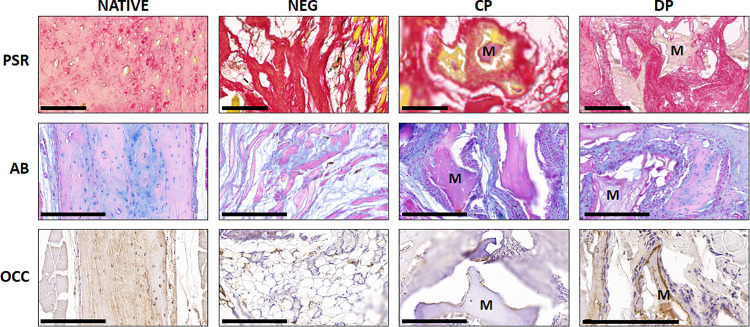
Histological analysis of animals grafted with different types of bone particles and controls using picrosirius red (PSR) and alcian blue (AB) histochemistry and osteocalcin (OCC) immunohistochemistry. Native: positive control of non-operated animals; NEG: negative control group of animals subjected to bone defect devoid of any bone particles; CP: animals subjected to bone defect filled with collagen-based particles; DP: animals subjected to bone defect filled with deproteinized particles. M: Mineralized structures. Scale bar: 200 µm.

**Fig 8:**
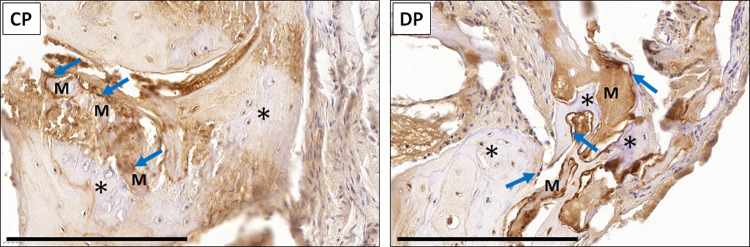
Histological analysis of bone regeneration associated to the grafted particles in the CP and DP groups of animals. Images correspond to osteocalcin immunohistochemistry analysis of defect areas close to the limits of the critical bone defect, after 2 months of follow-up. Grafted particles are highlighted with “M”, and asterisks (*) correspond to areas of native or regenerated bone tissue. Some cells showing positive osteocalcin staining signal that could correspond to differentiating osteoblasts are highlighted with blue arrows.

When the different samples grafted *in vivo* were analyzed using histochemistry and immunohistochemistry (Fig 7), we found some differences among groups. On the one hand, results of the PSR analysis showed that the normal bone tissue found in the Native group showed slightly positive PSR staining signal (+), suggesting the presence of a limited amount of collagen fibers within the bone extracellular matrix. In turn, the defect area found in NEG animals was strongly positive (+++), as it was also the case of the defect area corresponding to the CP group (+++). However, the DP group of animals showed a mild PSR staining signal (++) at the defect area. Interestingly, the mineralized particles found at the defect area in the CP group of animals were strongly positive (+++) for PSR, suggesting the presence of collagen in these particles. In contrast, mineralized particles found in the DP group were PSR negative (-). For AB histochemistry, our results revealed that both the Native and the NEG groups were slightly positive (+), whereas CP and DP showed a strong AB staining signal at the defect area (+++). On the other hand, immunohistochemical detection of OCC showed that this marker was present in the extracellular matrix of the Native bone, with a slightly positive signal (+), whilst this marker was negative in the NEG group (-) and could be detected on the surface of the mineralized particles found in the CP and DP groups with a mild staining signal (++). Analysis of the regeneration site in CP and DP groups demonstrated the presence of limited areas of bone that are apparently associated to the grafted CP and DP particles, without any detectable differences between both study groups (Fig 8). In addition, cells showing positive osteocalcin staining signal were found at these areas.

## Discussion

Several types of bone particles are widely used clinically to promote bone regeneration. In general, these particles are generated by decellularizing human or animal bones [27,28], and it has been demonstrated that the specific processing conditions used to fabricate these particles can significantly affect the structure and composition of the resulting product [29]. On the one hand, bone particles can be generated at low termperatures, which enables the bone matrix to preserve its collagen structure [12,13]. On the other hand, other types of bone particles can be generated using calcination or other biofabrication methods resulting in a hydroxyapatite-based bone matrix devoid of collagen [30]. As it has been reported that thermal treatment may significantly influence the biological, structural and chemical properties of biomaterials used in bone regeneration [31,32], determining which type of particles shows higher regenerative potential is in need. In general, preparation of particles at high temperatures (calcination) is associated with a complete removal of the organic components of the bone extracellular matrix, and a modification of the hydroxyapatite crystallin structure [33]. In this regard, the *ex vivo* and *in vivo* characterization analysis conducted in this study enabled us to contribute to a better characterization of the DP and CP bone particles, revealing differences between them that could be related to the distinct treatments they underwent during preparation.

When the morphology of CP and DP bone particles was analyzed, we found that both types of particles shared several similarities and were formed by a mixture of particles of different sizes. Although previous reports suggest that particles prepared at high temperatures could show higher compactation [34], we found that the porosity degree was similar for both types of materials, and the two types of particles consisted of a dense extracellular matrix with few empty spaces and cavities corresponding to the lacunae previously occupied by bone cells. The use of dense materials is very common in bone regeneration, although certain degree of porosity is always required [35].

One of the main requirements of biomaterials used clinically is biocompatibility, and analysis of this parameter is crucial to ensure the safe use of these products [36]. On this matter, when the biocompatibility of CP and DP particles was analyzed *ex vivo* using several complementary cell viability analysis methods, we observed that these particles were highly biocompatible to human gingival fibroblasts, although a transitory adaptation phase in which viability decreased temporarily was detected in the CP-DC group after 24h of follow-up. In general, cell viability remained over the 70% threshold established by ISO standards for assessing cell viability with biomaterials [37] at all times and in all conditions, which supports the high biocompatibility of both types of particles and predicts clinical biosafety, as previously demonstrated for other types of xenogeneic particles [38]. The fact that biocompatibility was assessed using an array of methods, including morphological analyses, metabolic tests, and structural damage detection, could contribute to increase the accuracy of the results obtained in the present work [39]. However, the fact that these analyses were carried out using gingival fibroblasts, and not osteoblast cells, requires the results to be viewed with care, and makes necessary the development of future studies using other types of cells.

To assess the impact of CP and DP particles on the bone regeneration process, we evaluated these biomaterials in an *in vivo* experimental model of mandibular bone damage previously described by other researchers [24]. This model allowed us to evaluate the effect of CP and DP particles on the regeneration process of a critical bone defect in the rat mandible [24]. It is important to note that this experimental model generated a bone lesion in an area predominantly consisting of compact bone tissue. Consequently, the results described in our work should be interpreted within this specific context.

When the functional effects of CP and DP particles were evaluated in this model of bone defect, we found that none of these particles was able to achieve complete regeneration of the critical bone defect after the established follow-up period of 2 months. However, both particles were able to partially reduce the size of the defect as compared to control animals devoid of any particles, and animals treated with CP particles showed a significant reduction of the mandibular defect and an increase in bone volume at the edges of the defect. These results are in agreement with other reports demonstrating that the use of bone filler particles could play an important role in bone tissue conduction [12,40,41], and with studies showing that the use of bone fillers containing collagen played an important role in increasing the amount of regenerative bone [41]. However, the critical size of the defect created in the animals included in our study was not completely regenerated, as reported for works in which a critical defect of similar size was generated in laboratory animals [24]. In this regard, it is important to note that the size of the defect plays an important role in bone regeneration, and previous studies using smaller defect size achieved positive bone regeneration [42]. Future studies should determine the regenerative potential of CP and DP particles applied to mandibular defects of smaller size, and if the regeneration of the large critical-size defect generated in the present work can be improved after longer follow-up periods.

Interestingly, the CT evaluation of animals grafted with the different particles showed the presence of radiopaque particulate structures at the defect site in DP animals. As the size of the defect was not significantly different to negative control animals, and in agreement with the results of the histological analyses carried out on DP animals, it is highly likely that these radiopaque structures correspond to the grafted bone filler particles, and not to greater bone neoformation at the recipient bed.

In line with the *ex vivo* results described above, our histological study of particles grafted *in vivo* contributed to confirming the high biocompatibility of the two types of particles analyzed here. The fact that our histological analyses revealed high biocompatibility of CP and DP, and tissues grafted with these particles were devoid from any detectable alterations, is in agreement with previously described results obtained in other animal models [43] and in human patients treated with these types of particles [30]. Despite the different composition of CP and DP, our study revealed that both types of biomaterials were highly biocompatible *in vivo*.

When the regenerative tissue was analyzed at the defect area, we found some differences among the study groups. In general, the use of both types of particles resulted in an increment of the connective tissue found at the defect area, as compared to the NEG group. This increment of connective tissue was associated with an increase in proteoglycans, confirmed by the AB staining analysis. Proteoglycans are essential components able of promoting cellular chemotaxis and differentiation during tissue regeneration, which can ultimately contribute to the mineralization process of bone regeneration by facilitating signaling transduction and maintaining stem cell homeostasis [44,45]. In fact, proteoglycans and glycosaminoglycans demonstrated to have capability to enhance osteoblast differentiation of undifferentiated human cells [46]. The increment of these molecules at the defect area confirms the regenerative potential of the CP and DP materials evaluated in the present work.

In addition, our histochemical analyses using PSR techniques showed that the regenerative tissue found in the CP group contained significant amounts of collagen, whereas DP animals had lower quantities of this type of fibers, suggesting that the mineralized structures found at the defect site of DP animals could correspond to the grafted deproteinized bone particles, and not to regenerated bone. As it was the case of proteoglycans, collagen fibers are essencial elements guiding the process of bone regeneration [47], and their presence are one of the main stimuli for bone mineralization during human development [48]. In fact, alterations in this fibrillar protein are typically associated with diverse bone diseases, such as osteogenesis imperfecta [49]. The fact that the composition of CP included native collagen fibers, could explain the higher synthesis of these fibrillar components of the extracellular matrix and might be associated with the more efficient effect of CP as compared to DP, devoid of these fibers. Related to this issue, we might hypothesize that CP could be able to attract extracellular matrix proteins with high affinity to bind to collagen, such as versican and other proteoglycans [50], which could provide CP particles with a basophilic pattern. However, our alcian blue histochemical analyses were not able to detect any differences between CP and DP particles grafted *in vivo*, which warrants the need for further studies. Most probably, matrix modulation could contribute to explain the results found in the present study and the different behaviour fo CP and DP grafted in laboratory animals.

Along with proteoglycans and collagen fibers, we determined the presence of osteocalcin, as an essential protein in the mineralization process [45,51]. In this regard, our findings align with those reported by Tapety et al. [52]. They observed an enhanced expression of osteocalcin when bone particles were utilized as filler materials, and concluded that these particles are able to contribute to the differentiation and function of local osteoblasts, thereby enhancing the bone regeneration process [52]. In our case, we found an increased expression of osteocalcin at the surface of the grafted filler particles, suggesting that these particles could induce local undifferentiated cells found at the defect site (probably, adipose-derived stem cells) [53] to synthesize this crucial bone protein, which is related to the process of bone regeneration and its expression is related to the mineralization process [54].

An interesting observation was the fact that most DP grafted at the defect site tended to remain *in situ*, at the grafted area, after the follow-up period. Most likely, this phenomenom could be related to the chemical composition of DP, which mostly consist of mineral inorganic material, which has been demonstrated to have lower remodelation rates by the recipient tissue and may require longer periods of time for a complete biointegration [55,56]. However, we may hypothesize that these particles could have important clinical applications in cases where augmentation of the volume of bone is required, such as ridge augmentation or sinus elevation, cases in which inorganic bovine bone has been used with good results [55,57]. In contrast, CP particles are likely more rapidly biodegraded and biointegrated, due to their organic and inorganic composition able to more efficiently mimic the native bone composition, as previously demonstrated [40,41]. The presence of multinucleated giant cells, indicative of bone particle remodeling, was observed in both cases, confirming that both types of particles are highly biomimetic.

In general, our results do not allow us to confirm if the grafted CP and DP particles have osteoconductive potential, as previously reported for other types of bone fillers [58]. However, we found limited areas of bone, probably corresponding to neoformed regenerative bone, associated with some of the grafted particles, that could indicate osteoconduction. The fact that these areas were not abundant could be related to the animal model used in the present work, as the rat angle of the mandible is mostly composed by a thin cortical bone [59], and it is well-known that cortical bone has lower regeneration potential than cancellous bone [60]. Although our results point out the possibility that CP and DP particles may have certain osteoconductive potential, future studies should be carried out to confirm or not this hypothesis. An interesting finding of our work is the presence of cells showing positive osteocalcin expression associated with the grafted particles and to the bone regeneration area at the limits of the defect. Although we cannot exclude other possibilities, it is likely that these cells correspond to differentiating osteoblasts that could be related to bone neoformation, as previously reported [61]. In this regard, previous studies using other types of biomaterials, such as biphasic calcium phosphate, were able to identify osteoblastogenesis associated to the grafted particles [62].

One unanswered question is the influence of the origin of the bone particles on the in vivo performance of CP and DP particles, as CP has porcine origin and DP are obtained from bovine sources. In this milieu, it is well known that bone structure and composition may differ among mammalian species [63]. In general, it has been demonstrated that the diameter of the Haversian canals is very similar in bovine and porcine bones, and this diameter is within the normal range of human bones [63]. Regarding the chemical composition, there is no consensus among researchers, and previous studies found that the Ca/P ratio of the bovine bone may range between 1.58 [64] and 1.98 [65], whereas the Ca/P ratio of the porcine bone ranges between 1.49 [66] and 1.71 [67], although these variations depend on the treatment applied to processing these particles [63]. In turn, the Ca/P ratio of the normal native human bone has been established between 1.78 and 2.55 [68]. The different composition of each type of particle and, especially, the methods applied to generate the bone filler particles, could play a significant role in determining the therapeutical properties of each type of particles, as the processing method could significantly modify the composition, thermostability, bone porosity and swelling degree of the particles [69]. According to our results, the use of collagen-based particles from porcine origin generated at low temperatures (CP) may offer the most adequate results in the animal model used in the present work, whereas particles subjected to calcination (DP), might have lower clinical usefulness in this animal model, at least, at the follow-up time analized here.

In summary, the comprehensive biocompatibility and functional analyses conducted in this study allowed us to shed light on the role of CP and DP in maxillofacial bone regeneration. In general, our results demonstrated that the particles employed were able to promote bone tissue regeneration, especially in the case of CP, although the critical size of the defect created in these animals could not be completely regenerated in any of the study groups during the established follow-up period. Future studies should determine the role of these particles in bone defects of smaller size. Although future studies should determine the clinical usefulness of these particles for the clinical treatment of patients with cleft palate, mandibular defects and other maxillofacial bone defects, involucring therapies with tissue engineered, our results could contribute to decision-making regarding the type of particles to be used in specific clinical situations.

## Supporting information

S1 File Video of a three-dimensional reconstruction of CP bone particles.
(AVI)

S2 File Video of a three-dimensional reconstruction of DP bone particles.
(AVI)

S1 Table Analysis of cell viability of human cells cultured in direct contact (DC) and indirect contact (IC) with collagen-based particles (CP) and deproteinized particles (DP) for 24, 48 and 72h, as determined by Live & Dead assay (LD) and free DNA quantification (DNA). For each study group, averages ± standard deviations are shown. The rows below correspond to the p values obtained for the statistical comparisons of CTR + vs. each study group. Statistically significant p values are highlighted with asterisks (*). CTR + : positive control of cells cultured without bone particles (live cells); CTR-: negative control of cells treated with 1% triton X-100 (dead cells).
(DOCX)

## References

[1] Kim N-H, Yang B-E, On S-W, Kwon I-J, Ahn K-M, Lee J-H, et al. Customized three-dimensional printed ceramic bone grafts for osseous defects: a prospective randomized study. Sci Rep. 2024;14(1):3397. doi: 10.1038/s41598-024-53686-w 38336901 PMC10858220

[2] Majidinia M, Sadeghpour A, Yousefi B. The roles of signaling pathways in bone repair and regeneration. J Cell Physiol. 2018;233(4):2937–48. doi: 10.1002/jcp.26042 28590066

[3] Liceras-Liceras E, Garzón I, España-López A, Oliveira A-C-X, García-Gómez M, Martín-Piedra M-Á, et al. Generation of a bioengineered autologous bone substitute for palate repair: an in vivo study in laboratory animals. J Tissue Eng Regen Med. 2017;11(6):1907–14. doi: 10.1002/term.2088 26449518

[4] Martín-Piedra MA, Alaminos M, Fernández-Valadés-Gámez R, España-López A, Liceras-Liceras E, Sánchez-Montesinos I, et al. Development of a multilayered palate substitute in rabbits: a histochemical ex vivo and in vivo analysis. Histochem Cell Biol. 2017;147(3):377–88. doi: 10.1007/s00418-016-1489-5 27600719

[5] Cota Quintero JL, Ramos-Payán R, Romero-Quintana JG, Ayala-Ham A, Bermúdez M, Aguilar-Medina EM. Hydrogel-Based Scaffolds: Advancing Bone Regeneration Through Tissue Engineering. Gels. 2025;11(3):175. doi: 10.3390/gels11030175 40136878 PMC11942283

[6] Mikos AG, Herring SW, Ochareon P, Elisseeff J, Lu HH, Kandel R, et al. Engineering complex tissues. Tissue Eng. 2006;12(12):3307–39. doi: 10.1089/ten.2006.12.3307 17518671 PMC2821210

[7] Thangavelu M, Kim D, Jeong YW, Lee W, Jung JJ, Song JE, et al. Enhancing Osteochondral Tissue Regeneration of Gellan Gum by Incorporating Gallus gallus var Domesticus-Derived Demineralized Bone Particle. Adv Exp Med Biol. 2020;1250:79–93. doi: 10.1007/978-981-15-3262-7_6 32601939

[8] Ashfaq R, Kovács A, Berkó S, Budai-Szűcs M. Developments in Alloplastic Bone Grafts and Barrier Membrane Biomaterials for Periodontal Guided Tissue and Bone Regeneration Therapy. Int J Mol Sci. 2024;25(14):7746. doi: 10.3390/ijms25147746 39062989 PMC11277074

[9] Araújo LK, Antunes GS, Melo MM, Castro-Silva II. Brazilian dentists’ perceptions of using bone grafts: an inland survey. Acta Odontol Latinoam. 2020;33(3):165–73. doi: 10.54589/aol.33/3/165 33523080

[10] Elboraey MO, Alqutaibi AY, Aboalrejal AN, Borzangy S, Zafar MS, Al-Gabri R, et al. Regenerative approaches in alveolar bone augmentation for dental implant placement: Techniques, biomaterials, and clinical decision-making: A comprehensive review. J Dent. 2025;154:105612. doi: 10.1016/j.jdent.2025.105612 39909139

[11] Ponte JS, Pérez-Guerrero JA, Aragão FA, Menezes YA, Melo MM, Castro-Silva II. Histomorphometric evaluation of human extraction sockets treated with autologous fibrin, sticky bone or biphasic calcium phosphate. Acta Odontol Latinoam. 2021;34(3):271–81. doi: 10.54589/aol.34/3/271 35088815 PMC10315085

[12] Falacho RI, Palma PJ, Marques JA, Figueiredo MH, Caramelo F, Dias I, et al. Collagenated Porcine Heterologous Bone Grafts: Histomorphometric Evaluation of Bone Formation Using Different Physical Forms in a Rabbit Cancellous Bone Model. Molecules. 2021;26(5):1339. doi: 10.3390/molecules26051339 33801547 PMC7958959

[13] Figueiredo A, Coimbra P, Cabrita A, Guerra F, Figueiredo M. Comparison of a xenogeneic and an alloplastic material used in dental implants in terms of physico-chemical characteristics and in vivo inflammatory response. Mater Sci Eng C Mater Biol Appl. 2013;33(6):3506–13. doi: 10.1016/j.msec.2013.04.047 23706240

[14] Boiko AA, Malanchuk VA, Myroshnychenko MS, Markovska OV, Shapkin AS, Marakushyn DI. Expression features of T-lymphocytes, B-lymphocytes and macrophages in the post-traumatic regenerate of the mandible rats under conditions of filling a bone defect with hydroxyapatite-containing osteotropic material and thymalin injecting the surrounding soft tissues. Pol Merkur Lekarski. 2024;52(2):171–7. doi: 10.36740/Merkur202402105 38642352

[15] Gönen ZB, Kemaloğlu CA, Gökdemir NS, Soylu E, Bolat D, Yay A. Mesenchymal Stem Cells-Derived Exosomes Combined With Bone Grafts Ameliorate Bone Regeneration in Mandibular Defects. J Craniofac Surg. 2025;10.1097/SCS.0000000000011087. doi: 10.1097/SCS.0000000000011087 39887216

[16] Guo J, Yao H, Chang L, Zhu W, Zhang Y, Li X, et al. Magnesium Nanocomposite Hydrogel Reverses the Pathologies to Enhance Mandible Regeneration. Adv Mater. 2025;37(2):e2312920. doi: 10.1002/adma.202312920 39385647 PMC11733717

[17] Fan Y-P, Lu J-F, Xu A-T, He F-M. Physiochemical characterization and biological effect of anorganic bovine bone matrix and organic-containing bovine bone matrix in comparison with Bio-Oss in rabbits. J Biomater Appl. 2018;33(4):566–75. doi: 10.1177/0885328218804926 30326803

[18] Li X, Lin S-C, Duan S-Y. The impact of deproteinized bovine bone particle size on histological outcomes in sinus floor elevation: a systematic review and meta-analysis. Int J Implant Dent. 2023;9(1):35. doi: 10.1186/s40729-023-00502-1 37782429 PMC10545653

[19] Vela-Romera A, Carriel V, Martín-Piedra MA, Aneiros-Fernández J, Campos F, Chato-Astrain J, et al. Characterization of the human ridged and non-ridged skin: a comprehensive histological, histochemical and immunohistochemical analysis. Histochem Cell Biol. 2019;151(1):57–73. doi: 10.1007/s00418-018-1701-x 30099600 PMC6328512

[20] Irastorza-Lorenzo A, Sánchez-Porras D, Ortiz-Arrabal O, de Frutos MJ, Esteban E, Fernández J, et al. Evaluation of Marine Agarose Biomaterials for Tissue Engineering Applications. Int J Mol Sci. 2021;22(4):1923. doi: 10.3390/ijms22041923 33672027 PMC7919481

[21] Rodriguez IA, Ferrara CA, Campos-Sanchez F, Alaminos M, Echevarría JU, Campos A. An in vitro biocompatibility study of conventional and resin-modified glass ionomer cements. J Adhes Dent. 2013;15(6):541–6. doi: 10.3290/j.jad.a29588 23593641

[22] Rodriguez MA, López-López MT, Durán JDG, Alaminos M, Campos A, Rodriguez IA. Cryopreservation of an artificial human oral mucosa stroma. A viability and rheological study. Cryobiology. 2013;67(3):355–62. doi: 10.1016/j.cryobiol.2013.10.003 24177233

[23] Campos F, Bonhome-Espinosa AB, Vizcaino G, Rodriguez IA, Duran-Herrera D, López-López MT, et al. Generation of genipin cross-linked fibrin-agarose hydrogel tissue-like models for tissue engineering applications. Biomed Mater. 2018;13(2):025021. doi: 10.1088/1748-605X/aa9ad2 29420310

[24] Awadeen MA, Al-Belasy FA, Ameen LE, Helal ME, Grawish ME. Early therapeutic effect of platelet-rich fibrin combined with allogeneic bone marrow-derived stem cells on rats’ critical-sized mandibular defects. World J Stem Cells. 2020;12(1):55–69. doi: 10.4252/wjsc.v12.i1.55 32110275 PMC7031757

[25] Hamad-Alrashid H, Muntión S, Sánchez-Guijo F, Borrajo-Sánchez J, Parreño-Manchado F, García-Cenador MB, et al. Bone Regeneration with Dental Pulp Stem Cells in an Experimental Model. J Pers Med. 2024;14(11):1075. doi: 10.3390/jpm14111075 39590567 PMC11595977

[26] Carriel VS, Aneiros-Fernandez J, Arias-Santiago S, Garzón IJ, Alaminos M, Campos A. A novel histochemical method for a simultaneous staining of melanin and collagen fibers. J Histochem Cytochem. 2011;59(3):270–7. doi: 10.1369/0022155410398001 21378281 PMC3201150

[27] Zhang X-H, Wang H, Cao Y, Liu L, Zhang Z-Q, Liu J-N, et al. Optimizing natural human-derived decellularized tissue materials for periodontal bone defect repair. Biochem Biophys Res Commun. 2025;748:151297. doi: 10.1016/j.bbrc.2025.151297 39818190

[28] Kandhari S, Khalid S, James A, Laverty DP. Bone grafting techniques and materials for implant dentistry. Br Dent J. 2023;235(3):180–9. doi: 10.1038/s41415-023-6113-1 37563385

[29] Terzioğlu P, Öğüt H, Kalemtaş A. Natural calcium phosphates from fish bones and their potential biomedical applications. Mater Sci Eng C Mater Biol Appl. 2018;91:899–911. doi: 10.1016/j.msec.2018.06.010 30033324

[30] Zhao R, Yang R, Cooper PR, Khurshid Z, Shavandi A, Ratnayake J. Bone Grafts and Substitutes in Dentistry: A Review of Current Trends and Developments. Molecules. 2021;26(10):3007. doi: 10.3390/molecules26103007 34070157 PMC8158510

[31] Lin FH, Liao CJ, Chen KS, Sun JS, Lin CY. Preparation of betaTCP/HAP biphasic ceramics with natural bone structure by heating bovine cancellous bone with the addition of (NH(4))(2)HPO(4). J Biomed Mater Res. 2000;51(2):157–63. doi: 10.1002/(sici)1097-4636(200008)51:2<157::aid-jbm3>3.0.co;2-r 10825214

[32] de Almeida GS, Suter LC, Pinto TS, Carra MGJ, da Silva Feltran G, de Moraes JF, et al. The Biological Properties of Co-Doped Monetite Are Influenced by Thermal Treatment. J Biomed Mater Res B Appl Biomater. 2025;113(2):e35531. doi: 10.1002/jbm.b.35531 39853958

[33] Lin FH, Liao CJ, Chen KS, Sun JS. Preparation of a biphasic porous bioceramic by heating bovine cancellous bone with Na4P2O7.10H2O addition. Biomaterials. 1999;20(5):475–84. doi: 10.1016/s0142-9612(98)00193-8 10204990

[34] Londoño-Restrepo SM, Jeronimo-Cruz R, Rubio-Rosas E, Rodriguez-García ME. The effect of cyclic heat treatment on the physicochemical properties of bio hydroxyapatite from bovine bone. J Mater Sci Mater Med. 2018;29(5):52. doi: 10.1007/s10856-018-6061-5 29721617

[35] Wei P, Zhou J, Xiong S, Yi F, Xu K, Liu M, et al. Chestnut-Inspired Hollow Hydroxyapatite 3D Printing Scaffolds Accelerate Bone Regeneration by Recruiting Calcium Ions and Regulating Inflammation. ACS Appl Mater Interfaces. 2024;16(8):9768–86. doi: 10.1021/acsami.3c17087 38349802

[36] Anderson JM. Future challenges in the in vitro and in vivo evaluation of biomaterial biocompatibility. Regen Biomater. 2016;3(2):73–7. doi: 10.1093/rb/rbw001 27047672 PMC4817327

[37] Chen I-C, Su C-Y, Lai C-C, Tsou Y-S, Zheng Y, Fang H-W. Preparation and Characterization of Moldable Demineralized Bone Matrix/Calcium Sulfate Composite Bone Graft Materials. J Funct Biomater. 2021;12(4):56. doi: 10.3390/jfb12040056 34698233 PMC8544512

[38] Dumitrescu CR, Neacsu IA, Surdu VA, Nicoara AI, Iordache F, Trusca R, et al. Nano-Hydroxyapatite vs. Xenografts: Synthesis, Characterization, and In Vitro Behavior. Nanomaterials (Basel). 2021;11(9):2289. doi: 10.3390/nano11092289 34578603 PMC8469747

[39] Stoddart MJ. Cell viability assays: introduction. Methods Mol Biol. 2011;740:1–6. doi: 10.1007/978-1-61779-108-6_1 21468961

[40] Nannmark U, Sennerby L. The bone tissue responses to prehydrated and collagenated cortico-cancellous porcine bone grafts: a study in rabbit maxillary defects. Clin Implant Dent Relat Res. 2008;10(4):264–70. doi: 10.1111/j.1708-8208.2007.00080.x 18241216

[41] Giuliani A, Iezzi G, Mazzoni S, Piattelli A, Perrotti V, Barone A. Regenerative properties of collagenated porcine bone grafts in human maxilla: demonstrative study of the kinetics by synchrotron radiation microtomography and light microscopy. Clin Oral Investig. 2018;22(1):505–13. doi: 10.1007/s00784-017-2139-6 28577053

[42] Kjalarsdóttir L, Dýrfjörd A, Dagbjartsson A, Laxdal EH, Örlygsson G, Gíslason J, et al. Bone remodeling effect of a chitosan and calcium phosphate-based composite. Regen Biomater. 2019;6(4):241–7. doi: 10.1093/rb/rbz009 31402983 PMC6683952

[43] van Houdt CIA, Ulrich DJO, Jansen JA, van den Beucken JJJP. The performance of CPC/PLGA and Bio-Oss® for bone regeneration in healthy and osteoporotic rats. J Biomed Mater Res B Appl Biomater. 2018;106(1):131–42. doi: 10.1002/jbm.b.33801 27889939

[44] Murshed M. Mechanism of Bone Mineralization. Cold Spring Harb Perspect Med. 2018;8(12):a031229. doi: 10.1101/cshperspect.a031229 29610149 PMC6280711

[45] Florencio-Silva R, Sasso GR da S, Sasso-Cerri E, Simões MJ, Cerri PS. Biology of Bone Tissue: Structure, Function, and Factors That Influence Bone Cells. Biomed Res Int. 2015;2015:421746. doi: 10.1155/2015/421746 26247020 PMC4515490

[46] Mathews S, Mathew SA, Gupta PK, Bhonde R, Totey S. Glycosaminoglycans enhance osteoblast differentiation of bone marrow derived human mesenchymal stem cells. J Tissue Eng Regen Med. 2014;8(2):143–52. doi: 10.1002/term.1507 22499338

[47] Lin X, Patil S, Gao Y-G, Qian A. The Bone Extracellular Matrix in Bone Formation and Regeneration. Front Pharmacol. 2020;11:757. doi: 10.3389/fphar.2020.00757 32528290 PMC7264100

[48] Selvaraj V, Sekaran S, Dhanasekaran A, Warrier S. Type 1 collagen: Synthesis, structure and key functions in bone mineralization. Differentiation. 2024;136:100757. doi: 10.1016/j.diff.2024.100757 38437764

[49] Yu H, Li C, Wu H, Xia W, Wang Y, Zhao J, et al. Pathogenic mechanisms of osteogenesis imperfecta, evidence for classification. Orphanet J Rare Dis. 2023;18(1):234. doi: 10.1186/s13023-023-02849-5 37559063 PMC10411007

[50] Chen D, Du Y, Llewellyn J, Bonna A, Zuo B, Janmey PA, et al. Versican binds collagen via its G3 domain and regulates the organization and mechanics of collagenous matrices. J Biol Chem. 2024;300(12):107968. doi: 10.1016/j.jbc.2024.107968 39510178 PMC11626796

[51] Martin-Piedra M-A, Gironés-Camarasa B, España-López A, Fernández-Valadés Gámez R, Blanco-Elices C, Garzón I, et al. Usefulness of a Nanostructured Fibrin-Agarose Bone Substitute in a Model of Severely Critical Mandible Bone Defect. Polymers (Basel). 2021;13(22):3939. doi: 10.3390/polym13223939 34833238 PMC8618832

[52] Tapety FI, Amizuka N, Uoshima K, Nomura S, Maeda T. A histological evaluation of the involvement of Bio-Oss in osteoblastic differentiation and matrix synthesis. Clin Oral Implants Res. 2004;15(3):315–24. doi: 10.1111/j.1600-0501.2004.01012.x 15142094

[53] Roato I, Belisario DC, Compagno M, Verderio L, Sighinolfi A, Mussano F, et al. Adipose-Derived Stromal Vascular Fraction/Xenohybrid Bone Scaffold: An Alternative Source for Bone Regeneration. Stem Cells Int. 2018;2018:4126379. doi: 10.1155/2018/4126379 29853912 PMC5949175

[54] Knabe C, Adel-Khattab D, Hübner W-D, Peters F, Knauf T, Peleska B, et al. Effect of silicon-doped calcium phosphate bone grafting materials on bone regeneration and osteogenic marker expression after implantation in the ovine scapula. J Biomed Mater Res B Appl Biomater. 2019;107(3):594–614. doi: 10.1002/jbm.b.34153 29770578

[55] Hallman M, Lundgren S, Sennerby L. Histologic analysis of clinical biopsies taken 6 months and 3 years after maxillary sinus floor augmentation with 80% bovine hydroxyapatite and 20% autogenous bone mixed with fibrin glue. Clin Implant Dent Relat Res. 2001;3(2):87–96. doi: 10.1111/j.1708-8208.2001.tb00236.x 11472655

[56] Hämmerle CHF, Jung RE, Yaman D, Lang NP. Ridge augmentation by applying bioresorbable membranes and deproteinized bovine bone mineral: a report of twelve consecutive cases. Clin Oral Implants Res. 2008;19(1):19–25. doi: 10.1111/j.1600-0501.2007.01407.x 17956571

[57] Artzi Z, Nemcovsky CE, Tal H. Efficacy of porous bovine bone mineral in various types of osseous deficiencies: clinical observations and literature review. Int J Periodontics Restorative Dent. 2001;21(4):395–405. 11519708

[58] Zhou H, Yang L, Gbureck U, Bhaduri SB, Sikder P. Monetite, an important calcium phosphate compound-Its synthesis, properties and applications in orthopedics. Acta Biomater. 2021;127:41–55. doi: 10.1016/j.actbio.2021.03.050 33812072

[59] Chin VKL, Shinagawa A, Naclério-Homem MdG. Bone healing of mandibular critical-size defects in spontaneously hypertensive rats. Braz Oral Res. 2013;27(5):423–30. doi: 10.1590/S1806-83242013000500006 24036980

[60] Liu L, Le PT, Stohn JP, Liu H, Ying W, Baron R, et al. Calorie restriction in mice impairs cortical but not trabecular peak bone mass by suppressing bone remodeling. J Bone Miner Res. 2024;39(8):1188–99. doi: 10.1093/jbmr/zjae104 38995944 PMC11337945

[61] Gao P, Liu C, Dong H, Li Q, Chen Y. TGF-β promotes the proliferation and osteogenic differentiation of dental pulp stem cells a systematic review and meta-analysis. Eur J Med Res. 2023;28(1):261. doi: 10.1186/s40001-023-01227-y 37501191 PMC10373408

[62] Lomelino R de O, Castro-Silva II, Linhares ABR, Alves GG, Santos SR de A, Gameiro VS, et al. The association of human primary bone cells with biphasic calcium phosphate (βTCP/HA 70:30) granules increases bone repair. J Mater Sci Mater Med. 2012;23(3):781–8. doi: 10.1007/s10856-011-4530-1 22201029

[63] Hillier ML, Bell LS. Differentiating human bone from animal bone: a review of histological methods. J Forensic Sci. 2007;52(2):249–63. doi: 10.1111/j.1556-4029.2006.00368.x 17316219

[64] Ratnayake JTB, Gould ML, Shavandi A, Mucalo M, Dias GJ. Development and characterization of a xenograft material from New Zealand sourced bovine cancellous bone. J Biomed Mater Res B Appl Biomater. 2017;105(5):1054–62. doi: 10.1002/jbm.b.33644 26968590

[65] Muñoz F, Haidar ZS, Puigdollers A, Guerra I, Padilla MC, Ortega N, et al. Efficient Hydroxyapatite Extraction from Salmon Bone Waste: An Improved Lab-Scaled Physico-Chemico-Biological Process. Molecules. 2024;29(17):4002. doi: 10.3390/molecules29174002 39274852 PMC11396111

[66] Kim S-H, Shin J-W, Park S-A, Kim YK, Park MS, Mok JM, et al. Chemical, structural properties, and osteoconductive effectiveness of bone block derived from porcine cancellous bone. J Biomed Mater Res B Appl Biomater. 2004;68(1):69–74. doi: 10.1002/jbm.b.10084 14689498

[67] Liu L, Li Q, Tang K, Yang L, Jian Y. Preparation and physicochemical properties of scaffold materials of heterogeneous deproteinized bone. Chin J Traumatol. 2007;10(1):59–62. 17229353

[68] Madeo B, De Vincentis S, Repaci A, Altieri P, Vicennati V, Kara E, et al. The calcium-to-phosphorous (Ca/P) ratio in the diagnosis of primary hyperparathyroidism and hypoparathyroidism: a multicentric study. Endocrine. 2020;68(3):679–87. doi: 10.1007/s12020-020-02276-7 32236819

[69] de Lopes MS, de Souza FFP, Mattos ALA, Gomes MJP, Castro-Silva II. Influence of Hydrogen Peroxide on Composition, Thermostability, Porosity and Swelling of Collagen Matrices of Demineralized Porcine Cortical Bone. Braz arch biol technol. 2024;67. doi: 10.1590/1678-4324-2024230742

