# Supplementary material for: A comprehensive analysis of two types of xenogeneic bone particles for use in maxillofacial bone regeneration therapies
Source: PLoS One. 2025 May 19;20(5):e0323754. doi: 10.1371/journal.pone.0323754 (PMC12088006; doi:10.1371/journal.pone.0323754)
Supplement: S1 Table — Analysis of cell viability of human cells cultured in direct contact (DC) and indirect contact (IC) with collagen-based particles (CP) and deproteinized particles (DP) for 24, 48 and 72h, as determined by Live & Dead assay (LD) and free DNA quantification (DNA). For each study group, averages ± standard deviations are shown. The rows below correspond to the p values obtained for the statistical comparisons of CTR + vs. each study group. Statistically significant p values are highlighted with asterisks (*). CTR + : positive control of cells cultured without bone particles (live cells); CTR-: negative control of cells treated with 1% triton X-100 (dead cells). (DOCX) [file pone.0323754.s003.docx]

|  | **LD** | | | **DNA** | | |
| --- | --- | --- | --- | --- | --- | --- |
|  | **24h** | **48h** | **72h** | **24h** | **48h** | **72h** |
| **CTR+** | **100±0.74** | **100±0.53** | **100±0.08** | **100±1.18** | **100±1.78** | **100±1.80** |
| **CTR-** | **0±0** | **0±0** | **0±0** | **0±7.42** | **0±19.62** | **0±8.07** |
| **CP-IC** | **99.99±1.25** | **98.78±2.00** | **100.02±0** | **98.61±1.12** | **100.50±2.64** | **101.18±1.32** |
| **CP-DC** | **88.83±9.79** | **93.18±4.63** | **96.82±2.09** | **98.81±1.68** | **100.65±1.55** | **101.38±1.50** |
| **DP-IC** | **100.06±1.10** | **99.36±1.49** | **100.01±0.06** | **103.21±1.01** | **105.02±1.66** | **105.75±1.07** |
| **DP-DC** | **90.2±8.56** | **95.42±4.44** | **97.31±1.00** | **104.30±1.48** | **105.73±0.91** | **105.76±1.22** |
| **CTR+vs.CTR-** | **p<0.0001*** | **p<0.0001*** | **p<0.0001*** | **p<0.0001*** | **p<0.0001*** | **p<0.0001*** |
| **CTR+vs.CP-IC** | **p=0.9999** | **p=0.9999** | **p=0.9999** | **p=0.9999** | **p=0.9999** | **p=0.9999** |
| **CTR+vs.CP-DC** | **p=0.0007*** | **p=0.0289** | **p=0.2463** | **p=0.9999** | **p=0.9999** | **p=0.9999** |
| **CTR+vs.DP-IC** | **p=0.9999** | **p=0.9999** | **p=0.9999** | **p=0.2463** | **p=0.0593** | **p=0.0593** |
| **CTR+vs.DP-DC** | **p=0.0032** | **p=0.1212** | **p=0.4975** | **p=0.1213** | **p=0.0593** | **p=0.0593** |
